# Evaluation of a Retrieval-Augmented Generation–Based Large Language Model for Evidence-Based Herb and Supplement Information in Cancer Care

**DOI:** 10.2196/86073

**Published:** 2026-07-09

**Authors:** Yen-Nien Hou, Jyothirmai Gubili, Pulkit Jain, Chun Sing Lam, Avijit Chatterjee, Jun J Mao

**Affiliations:** 1 Integrative Medicine and Wellness Service, Memorial Sloan Kettering Cancer Center, 321 E 61st Street, 4th Fl., New York, NY, 10065, United States, 1 6466088550; 2 Digital, Informatics and Technology Solutions (DigITs), Artificial Intelligence & Machine Learning, Memorial Sloan Kettering Cancer Center, New York, NY, United States

**Keywords:** herbs, dietary supplements, cancer, health information, artificial intelligence

## Abstract

Our study describes the development and evaluation of a retrieval-augmented generation–based large language model to improve the quality of responses to provider questions about herbs and dietary supplements.

## Introduction

Patients with cancer increasingly seek herbs and dietary supplements for symptom relief [1]. However, concurrent use of supplements with cancer treatment can lead to harmful adverse events and herb-drug interactions [2]. Additionally, many oncology providers have limited knowledge/training to counsel patients [3]. There is also a growing amount of misinformation about cancer treatments and exaggerated benefits of natural products for improving outcomes including curing cancer [4].

The *About Herbs* app—developed by the Integrative Medicine Service at Memorial Sloan Kettering Cancer Center (MSK)—delivers content from the *About Herbs* database that contains the latest research on 317 entries including herbs, minerals, and vitamins [5]. Currently, the app uses a keyword-based search system, which is inadequate to answer complex queries typically asked by providers. This can hinder their communication with patients on this important topic.

Since 2022, large language models (LLMs) are being increasingly used to obtain health-related information. While LLMs outperform online searches, they are prone to producing inaccurate responses or hallucinations [6], which can be especially harmful in oncology settings. Retrieval-augmented generation (RAG) is a technique that can improve the accuracy of responses by integrating the LLM with a searchable knowledge base [7]. To enhance the search functionality of the *About Herbs* app, we developed and evaluated a RAG-based LLM grounded in the *About Herbs* database.

## Methods

### Overview

We selected 26 monographs with extensive clinical data, which included commonly queried herbs/supplements in oncology practice at MSK, as the knowledge source for developing the RAG-based LLM (*AboutHerbsAI*): active hexose correlated compound, aloe vera, ashwagandha, astragalus, biotin, Boswellia, broccoli sprouts, calcium, cannabis, carnitine, coenzyme Q10, dong quai, flaxseed, folate, glucosamine, glutamine, green tea, licorice, melatonin, omega-3, probiotics, psilocybin, soy, turmeric, vitamin C, and vitamin D.

Development of *AboutHerbsAI* is described in Multimedia Appendix 1 [7,8].

### Evaluation of *AboutHerbsAI* versus Current State-of-the Art LLMs

We developed 5 commonly asked questions (see Multimedia Appendix 1) surrounding herb/supplement use in cancer care, focusing on safety and dietary precautions, symptom management, supportive benefits during treatment, expected effects, and potential herb–drug interactions. These domains reflect common patient counseling considerations.

To compare with *AboutHerbsAI,* we chose 9 general-purpose LLMs available in April 2025 from 3 major vendors—OpenAI (gpt-35-turbo-0613, gpt-4-turbo-2024-04-09, and gpt-4o-20240513); Anthropic (anthropic.claude-3-opus-20240229-v1:0, anthropic.claude-3-haiku-20240307-v1:0, and anthropic.claude-3‐5-sonnet-20240620-v1:0); and Meta (meta.llama3-1-8b-instruct-v1:0, meta.llama3-1-70b-instruct-v1:0, and meta.llama3-1-405b-instruct-v1:0)—to reflect the systems most likely to be accessed by clinicians (LLM settings are in Multimedia Appendix 1). Based on the QUEST (quality of information, understanding and reasoning, expression style and persona, safety and harm, and trust and confidence) framework for human evaluation of LLMs in health care as well as other RAG evaluation frameworks [9] and expert input, we arrived at 3 metrics to capture the quality of answers: (1) completeness (“Does the answer include all the pertinent information?”), (2) correctness (“Does the answer include false or true information?”), and (3) conciseness (“Does the answer include any non-pertinent information?”). The answers were rated on a binary scale of *true* or *false*.

We randomly distributed the 26 monographs among 3 reviewers. They independently rated the answers to the 5 questions for all LLMs for the set of monographs assigned to them. The reviewers were not aware of which LLM each answer was coming from. We compiled the reviewer ratings and aggregated them to derive our final score for a particular LLM on a given metric. Interrater reliability assessment details are in Multimedia Appendix 1.

To compare performance between *AboutHerbsAI* and other LLMs, a chi-square test was used to assess whether the proportion of positive ratings differed significantly between models for each metric.

## Results

**Figure 1.**
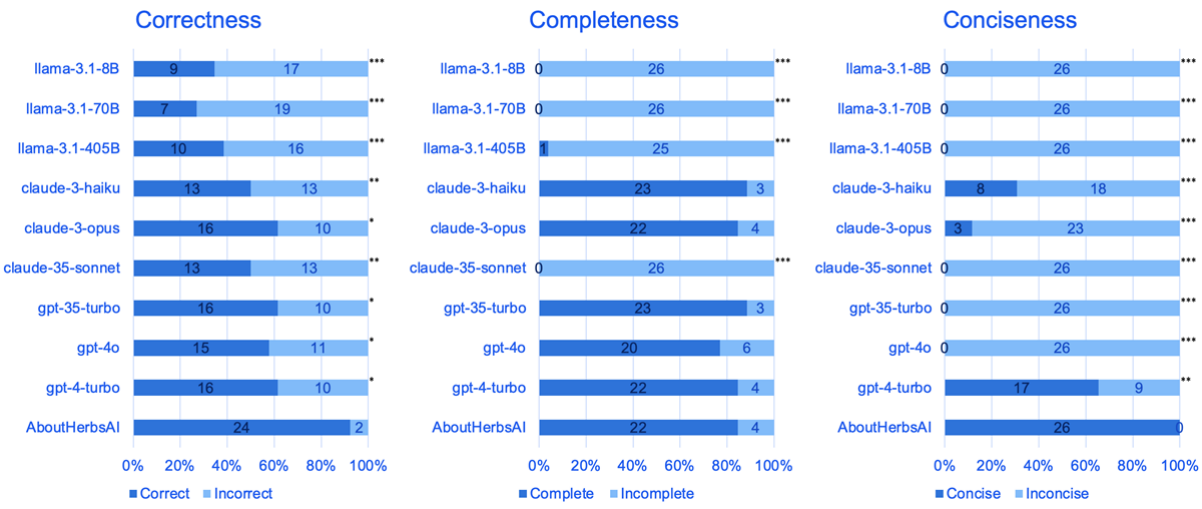
Comparison of RAG-based LLM (*AboutHerbsAI*) with 9 general-purpose LLMs available in April 2025 from 3 major vendors (OpenAI, Anthropic, and Meta). LLM: large language model; RAG: retrieval-augmented generation. *P≤.05; **P≤.01; ***P≤.001.

In total, we evaluated 1300 question-answer pairs across 5 expert-curated questions, 26 monographs, and 9 LLMs and *AboutHerbsAI*. Interrater agreement was substantial for across rater pairs (κ=0.66‐0.74). Our model was superior to the 9 LLMs in both correctness and conciseness of responses with no significant differences in completeness (Figure 1).

### Correctness

*AboutHerbsAI* provided correct responses in 92.3% (24/26) cases compared to the leading LLMs (Claude-3-opus, GPT-35-turbo, and GPT-4-turbo) at 61.5% (16/26; *P*=.02). It also generated significantly more correct responses than other LLMs tested (range: 26.9% [7/26] to 57.7% [15/26]; all *P*<.02).

### Completeness

*AboutHerbsAI* provided complete responses in 84.6% (22/26) cases compared to the leading LLMs (Claude-3-haiku and GPT-35-turbo) at 88.5% (23/26) with no statistically significant differences (*P*>.99). It also generated significantly more complete responses than Llama-3.1-8B, Llama-3.1-70B, Llama-3.1-405B and Claude-35-sonnet (range: 0% [0/26] to 3.8% [1/26]; all *P*<.001).

### Conciseness

*AboutHerbsAI* provided concise responses in 100% (26/26) cases compared to the leading LLM (GPT-4-turbo) at 65.4% (17/26; *P*=.003). It also generated significantly more concise responses than all other LLMs (range: 0% [0/26] to 30.8% [8/26]; all *P*<.001).

## Discussion

This study found that a RAG-based LLM substantially improved correctness and conciseness of responses to common questions about herbs and dietary supplements, when compared with 9 general-purpose LLMs, but slightly underperformed in completeness. We consider this acceptable given the benefit of grounding responses in an evidence-based database rather than open internet sources, which are prone to misinformation.

These results align with prior concerns about LLM hallucinations [6] and complementary and alternative medicine misinformation [4] and support our approach as a proof-of-concept study for integrating evidence-grounded LLM assistance [7] to improve the quality and accuracy of responses. This study is timely as oncology care is becoming increasingly digitized. An important limitation is the confinement of our model to a curated database that limits response breadth, especially when newer evidence is not yet included. Second, its performance was assessed in a controlled setting with limited content and predefined questions. Therefore, generalizability, and real-world queries—particularly those using vague or nonmedical/nontechnical language (eg, “hangover”)—may pose challenges [10].

Future work will include system enhancements and evaluation of the clinical/patient-facing impacts of *About Herbs*. Database curation will involve regular ingestion of the latest literature to ensure timeliness. To support patients with varying health literacy needs, the app will offer a configurable layperson-oriented version to deliver information. Potential clinical implementations include embedding the RAG-based LLM within the *About Herbs* app to help physicians deliver evidence-based recommendations to minimize risks associated with the concomitant use of supplements during cancer treatment. Evaluation will involve assessing clinical significance, including effects on herb-related counseling, documentation, and safety, alongside integration into electronic medical records and clinic workflows.

## Supplementary material

10.2196/86073 Multimedia Appendix 1 Methods, including development and evaluation of *AboutHerbsAI*, LLM settings, and the interrater reliability assessment.

## Abbreviations

LLM: large language model
MSK: Memorial Sloan Kettering Cancer Center
QUEST: quality of information, understanding and reasoning, expression style and persona, safety and harm, and trust and confidence
RAG: retrieval-augmented generation
