# Supplementary material for: Evaluation of a Retrieval-Augmented Generation–Based Large Language Model for Evidence-Based Herb and Supplement Information in Cancer Care
Source: JMIR Cancer. 2026 Jul 9;12:e86073. doi: 10.2196/86073 (PMC13349407; doi:10.2196/86073)
Supplement: Multimedia Appendix 1 [file cancer-v12-e86073-s001.docx]

**Methods**

**Development of *AboutHerbsAI***
The 26 monographs were split into smaller chunks ensuring the relevant information contained in each section stayed together [17]. We experimented with different chunk sizes, overlaps, and separators as well as the number of chunks returned per query to determine the optimal setup. The text-embedding-ada-002 model was used to generate text embeddings for these chunks that were stored in a vector database. For every question asked, we found the closest embeddings to the question in the vector database to use as context for an LLM. For retrieval, we implemented a dynamic threshold because the monographs vary in the quantity of information [1]. Several rounds of prompt engineering were conducted to find the best prompt for our use case [2]. Finally, we fed the retrieved monograph chunks to the OpenAI text-davinci-003 model along with user query and prompt to generate answers.

**Evaluation of *AboutHerbsAI***

The five questions we developed for this study were: 1. Are there any specific dietary restrictions or considerations cancer patients should be aware of when using [herb/supplement]? 2. Is [herb/supplement] recommended for managing common side effects of cancer treatment, such as nausea, fatigue, or immune suppression? 3. Is [herb/supplement] known to support cancer patients during treatment, and what evidence supports its use? 4. What symptoms or side effects I can find helpful using [herb/supplement]? and 5. What drugs should be avoided when taking [herb/supplement] due to potential herb-drug interactions?

**LLM Settings**

Across the LLMs, we used identical inference settings to ensure comparability. No system prompts or additional prompt engineering were applied; each model received the same set of questions verbatim. We standardized decoding parameters to temperature = 1.0 and max_tokens = 512 for every model.

**Inter-rater Reliability Assessment**

To assess interrater reliability, reviewers additionally evaluated a subset of monographs originally assigned to another reviewer, creating overlapping subsets. Ratings from overlapping evaluations were used solely to quantify agreement between reviewers and were not adjudicated or merged to determine final outcome labels. Disagreements between reviewers were therefore retained as independent judgments and reflected only in the inter-rater reliability analysis. Interrater reliability was assessed using pairwise Cohen’s κ on overlapping subsets of items.

**References**

1. Lewis P, Perez E, Piktus A, Petroni F, Karpukhin V, Goyal N, et al. Retrieval-Augmented Generation for Knowledge-Intensive NLP Tasks. 34th Conference on Neural Information Processing Systems (NeurIPS 2020); Vancouver, Canada2020.

2. Meskó B. Prompt Engineering as an Important Emerging Skill for Medical Professionals: Tutorial. J Med Internet Res. 2023 Oct 4;25:e50638. PMID: 37792434. doi: 10.2196/50638.
